# The impact of COVID-19 pandemic on colorectal cancer patients at an NHS Foundation Trust hospital-A retrospective cohort study

**DOI:** 10.1016/j.amsu.2021.103182

**Published:** 2021-12-15

**Authors:** Omotara Kafayat Lesi, Ebuwa Igho-Osagie, Sarah-Jane Walton

**Affiliations:** a Mid and South Essex NHS Foundation Trust, Basildon and Thurrock University Hospitals, Essex, United Kingdom; b Tufts School of Medicine, Boston, MA, United States

**Keywords:** Colorectal cancer, COVID-19, Cancer progression

## Abstract

**Introduction:**

Current NHS guidelines recommend that treatment of colorectal patients referred through the two-week wait referral system should occur within sixty two days from the date of referral. The COVID-19 pandemic which started in March 2020 has however led to significant delays in the delivery of health services, including colorectal cancer treatments. This study investigates the effects of delayed colorectal cancer treatments during the COVID pandemic on disease progression.

**Methods:**

A retrospective chart review of 107 patients with histologically confirmed diagnosis of colorectal cancer was conducted. The occurrence of cancer upstaging after initial diagnosis was assessed and compared between patients with treatment delays and patients who received treatments within the period recommended by NHS guidelines. A logistic regression was performed to evaluate the association between treatment delays beyond 62 days and cancer upstaging.

**Results:**

The median age of the cohort was 71.2 years and 64.5% of the patients were over 65 years. Treatment delays were observed in 53.3% of reviewed patients. Patients with treatment delays received cancer treatments 95.8 (31.0) days on average after referral, compared to 46.3 (11.5) days in patients who experienced no treatment delays (p-value<0.0001). 38.6% of patients with treatment delays experienced cancer upstaging by the time of treatment, compared to 20% in the non-delay group (p-value = 0.036). Patients who received treatment after sixty two days from date of referral were 3.27 times more likely to experience colorectal cancer upstaging compared to those who received timely treatments.

**Conclusion:**

Although an effective response to the Covid-19 pandemic requires the reallocation of healthcare resources, there is a need to ensure that treatments and health outcomes of patients with chronic diseases such as colorectal cancer continue to be prioritized and delivered in timely fashion.

## Highlights

• The COVID-19 pandemic led to significant delays in the delivery of health services, including colorectal cancer treatments in the United Kingdom.
• Effect of delayed colorectal cancer treatments on upstaging of tumours was reviewed.
• Patients who received treatment after sixty-two days from date of referral were 3.27 times more likely to experience colorectal cancer upstaging compared to those who received timely treatments.

## 1 Introduction

Colorectal cancer is the fourth commonest cancer in the United Kingdom [1]. In 2017, it accounted for eleven percent of all new cancer cases with an estimated 42,100 new cases. It is projected to be prevalent in 74 per 100,000 UK citizens buy 2035 [1]. About 16,600 colorectal cancer deaths occur annually in the UK with mortality rates highest in those aged over 90 years [1] and more than half of the cases (52–56%) are diagnosed at a late stage (stage III or IV) [1]. Although colorectal cancer incidence rates in the United Kingdom are highest in the very elderly (85–89 years), its incidence in younger people is rising [2].

Patients typically present in one of the following ways: asymptomatic patients detected by routine screening; symptomatic patients with suspicious symptoms and signs, and patients presenting as an emergency with intestinal obstruction, perforation, or in rare cases, acute gastrointestinal bleeding [3]. Other presenting symptoms of colorectal cancer include change in bowel habits, rectal bleeding, rectal mass/abdominal mass, iron deficiency anaemia, and abdominal pain [3]. Presenting symptoms typically indicate tumour location. Changes in bowel habits more commonly occurs with tumours occurring in the left side of the abdomen; bleeding per rectum is more often seen in tumours affecting the rectosigmoid, while iron deficiency anaemia is more associated with right-sided tumours. Rectal cancers may present with rectal pain, tenesmus and reduced caliber of stools.

Colorectal cancer prognosis is associated with presenting symptoms and survival is strongly associated with the stage of cancer at diagnosis [4]. Patients with obstruction or perforation tend to have a worse prognosis independent of stage [5,6]. Bleeding per rectum as the initial symptom is linked with a lower stage of colorectal cancer and mild anaemia with a haemoglobin of 10–12.9 g/dl is associated with a higher cancer staging [7]. Five-year survival rates in the UK are noted to be 93.2% for stage I, 84.7% for stage IIa, 72.2% for stage IIb, 83.4% for stage IIIA, 64.1% for stage IIIb, 44.3% for stage IIIc, and 8.1% for stage IV [8]. Survival rates in the UK are lower compared to other countries. The five year survival rate for colon cancer in England is 51% and 52% for men and women respectively compared to an average of 56% in both sexes across Europe [9].

Surgical resection is the central component of colorectal cancer treatment while systemic chemotherapy and local pelvic radiotherapy are important adjuvant or neo-adjuvant treatment modalities [10]. The aim of surgery for colorectal cancer is curative and entails complete excision of the tumour along with its major vascular pedicle and lymphatic supply. Resection is performed through open, laparoscopic or robotic means. Chemotherapy is usually given post-operatively when patients have stage III tumours (these are tumours with spread to lymph nodes). Pre-operative chemotherapy and radiotherapy is the usual modality for patients with locally advanced rectal cancer [11].

### 1.1 Effect of COVID pandemic

Covid-19 was declared a global pandemic by the World Health Organization (WHO) in March 2020 [12]. The high rate of transmission, severe respiratory and non-respiratory complications of the disease has placed a huge burden on health systems’ globally. Healthcare, political and public health resources across the globe have been reprioritized to respond to the disease. In the UK, the pressure of Covid-19 on National Health Service (NHS) has led to the postponement of millions of elective surgery cases and a backlog of treatment [13]. An estimated 650,000 people (22%) with cancer experienced disruption to their cancer treatment or care in the form of postponement of cancer surgery and/or delivery of chemotherapy as a result of COVID-19 [14]. Cancer treatments in the UK were impacted by Covid-19 due to the deployment of staff from surgical units to medical wards and intensive care units to manage Covid-19 complications [13].

The NHS and other UK healthcare decision makers have made recommendations for centres delivering colorectal cancer treatments during the pandemic [15,16]. Surgeons were advised to reduce the risk of Covid-19 spread by replacing aerosol-generating procedures like laparoscopic and robotic surgeries with open abdominal surgeries [17]. The formation of stomas was also recommended over performing a bowel anastomosis to reduce the risk of anastomotic leaks (potential need for intensive care unit or return to the theatre) [17]. The use of short-course radiotherapy with a delay in surgery was also advised for patients with advanced disease to reduce the risk of patients being exposed to SARS-CoV-2 infection in the hospitals [18].

### 1.2 Association between treatment delays and health outcomes

The desired targets for treatment of cancer cases in England, published in the National Health Service(NHS) Cancer plan of September 2000 [19], include no more than 62 days delay between the date the hospital receives an urgent referral for suspected cancer and the start of treatment [19]. Also, the NHS recommends that there should be no more than 31 days delay between the appointment at which the patient and the clinician agree to the treatment plan and the start of treatment [19]. These specific cancer waiting times were published as part of a ten-year programme to reform cancer services and improve cancer survival in the United Kingdom. However, evidence of impact of treatment delays on outcomes linked with survival such as disease progression (cancer upstaging), and survival itself, is uncertain [20]. Several studies have demonstrated little or no impact of treatment delays on colorectal cancer outcomes [[21], [22], [23], [24], [25], [26], [27],[27], [27], [28], [29]]. Simunovic et al. [28] demonstrated that delays of more than six weeks from the first diagnostic test to surgical admission had no impact on the risk of operative mortality or disease-specific survival [28]. Similarly, Murchie et al.(26) and Terhaar sive Droste et al.(29) studies showed that longer diagnostic and therapeutic delays were not associated with poorer survival. A meta-analysis examining associations between diagnostic and therapeutic delays on patient survival failed to show an association between delay and survival [24].

On the other hand, researchers have also pointed to an association between treatment delays of colorectal cancer and colorectal cancer outcomes. Langenbach et al. [30] demonstrated that an increased interval in time from first symptoms until surgical therapy was associated with an increased probability of advanced tumour stage and a decreased probability of survival. Rowe-Jones and Aylett's [31] review of 200 patients with colorectal cancer in a London hospital concluded that treatment delays lead to a more advanced stage of cancer by the time of treatment, and this worsened prognosis accordingly. Iverson et al. [32] prospective's study of 740 patients with colorectal cancer showed that a delay of 60 days from the date of onset of symptoms was significantly linked with poorer long term outcomes in symptomatic rectal cancer patients. A 2021 systematic review and meta-analysis by Whittaker et al. [33] confirmed the importance of not delaying treatment as results indicated that delays over 4 weeks led to poorer outcomes.

Given the uncertainty of evidence on the effects of treatment delays on colorectal cancer outcomes, this study sought to investigate the association between delays in neo-adjuvant therapy (such as chemotherapy or radiotherapy) and surgery and disease progression in patients seen at one of the hospitals in the UK.

## 2 Methods

### 2.1 Study design

This study was a bi-centre, retrospective chart review study conducted using patient registry data of 107 patients with colorectal cancer seen between January 1, 2020 to December 31, 2020 at a hospital in the United Kingdom.

We defined treatment delay according to NHS guidelines as greater than 62 days (2 months) from the date the specialist hospital receives a referral for suspected cancer from the general practitioner (GP) and the start of neoadjuvant chemoradiotherapy/surgery for confirmed cancer [19].

Following expert consultation, cancer upstaging in this study was defined as progression from a lower stage to a higher number stage i.e. from stage I to II, stage II to IV, stage I to III or from stage II to III using the combined American Joint Committee on Cancer (AJCC) or Union for International Cancer Control (UICC), eighth edition, 2017 [34].

The work has been reported in line with the STROCSS criteria [35].

### 2.2 Participants

Study participants were patients from one of the NHS Foundation trusts who were referred through the two week-wait referral system (in which patients with suspected cancer must be seen by a specialist within two weeks) [19]. Participants were adults 18 years and older with a confirmed histological diagnosis of colorectal cancer through colonoscopy or flexible sigmoidoscopy.

All cases were discussed at the colorectal multidisciplinary team (MDT) meeting for curative intent with surgery and/or chemoradiotherapy. The TNM/AJCC staging for colorectal cancer was documented in the MDT meeting and was derived from pre-treatment radiological images. Patients with stage IV cancer (metastatic cancer), patients prescribed palliative treatment or best supportive treatment as well as those unfit for treatment were excluded from the study. Patients diagnosed with colorectal cancer through another route other than the two week referral system, e.g. those who presented as an emergency and urgent upgrade were excluded from the study.

### 2.3 Study variables

Demographic, behavioural, and clinical variables were extracted from the clinical records of study participants and included as covariates in the logistic regression analyses. Extracted variables were age, gender, body mass index (BMI), smoking status and alcohol use. Clinical variables were the date of the decision to refer, date of diagnosis, date of multi-disciplinary team (MDT) meeting, Tumour, node, metastases (TNM) or American Joint Committee on Cancer (AJCC) stage of cancer, location of tumour and date of first treatment (surgery or preoperative chemoradiotherapy). Tumours were grouped based on their locations. Patients with caecal, ascending colon, hepatic flexure, and transverse colon cancers were grouped as right colon cancers. Those with splenic flexure, descending colon, and sigmoid colon cancers were grouped as left colon cancers while those with rectal and recto-sigmoid cancers were grouped as rectal cancers. The date of first treatment was the date the patients had their surgery or commenced their pre-operative chemotherapy or radiotherapy. The date from the day of referral to the day of first treatment was calculated and more than 62 days was determined as delay in treatment. The type of surgical procedure done was documented as either open or laparoscopic surgery. The post-operative diagnosis for those who had surgery was derived from the histopathological results using TNM/AJCC staging system. Those that had pre-operative chemoradiotherapy had repeat radiological scans after and the TNM staging was derived from the images. Pre-treatment staging was then compared to post-treatment staging and patients that had upstaged were those who had progressed from a lower to a higher stage.

### 2.4 Data analysis

Multivariable logistic regression was used to examine the relationship between treatment delay and the likelihood of cancer upstaging. Co-variates included in the logistic model were age, gender, smoking status, BMI, and alcohol consumption. Using the G-power calculator [36] we estimated that the study had a power of 89% to detect an odds ratio of ≥2 given a sample size of 107 patients and statistical significance of p < 0.05. All analyses were performed with Stata statistical package.

## 3 Results

The mean age of participants was 69.4 ± 12.0 years (median = 71.2 years). Sixty nine patients (64.5%) were over sixty years old. Fifty (46.7%) were males, and fifty patients had a history of past or current smoker (ever smoked). 49.5% of the patients had stage III cancer according to the AJCC classification. The most common site for colorectal cancer was the right side (caecal, ascending, right, hepatic flexure, and transverse colon cancers) accounting for 44% of the patients. Left-sided tumours (splenic flexure, descending colon, and sigmoid colon tumours) accounted for 23% and rectal cancer (rectosigmoid and rectal tumours) constituted 31% (Table 1).

**Table 1:** Baseline clinical and demographic variables.

Demographic Variable Mean (SD) or n (%)	Total (n = 107)	No delay in treatment (n = 50)	Delay in treatment (n = 57)
Age (years)	69.4 (12.0)	67.0 (11.9)	71.5 (11.8)
≥ 65 years	69 (64.5%)	29 (42%)	40 (58%)
Male	50 (46.7%)	21 (42%)	29 (58%)
BMI	26.9 (5.3)	27.0 (5.4)	26.4 (5.2)
Obese	20 (21.5%)	9 (19.2%)	11 (23.9%)
Ever-smoked	50 (47.6%)	19 (38%)	31 (52%)
Alcohol use	64 (62.1%)	34 (53.1%)	30 (46.9%)
Disease pre-treatment staging			
**Stage 0**	4 (3.7%)	0 (0.0%)	4 (100.0%)
**Stage 1**	25 (23.4%)	8 (32.0%)	17 (68.0%)
**Stage 2**	35 (23.4%)	14 (56.0%)	11 (44.0%)
**Stage 3**	53 (49.5%)	28 (52.8%)	25 (47.2%)
Disease Location			
**Right Colon**	47 (43.9%)	21 (44.7%)	26(55.3%)
**Left Colon**	25 (23.4%)	14 (56%)	11 (44.0%)
**Rectum**	33 (30.8%)	15 (45.5%)	18 (54.5%)
**Synchronous**	2 (1.9%)	0 (0.0%	2 (100%)
Number of days between referral and treatment (mean)	72.7 (34.5	46.3 (11.5)	95.8 (31.0)

**Fig. 1:**
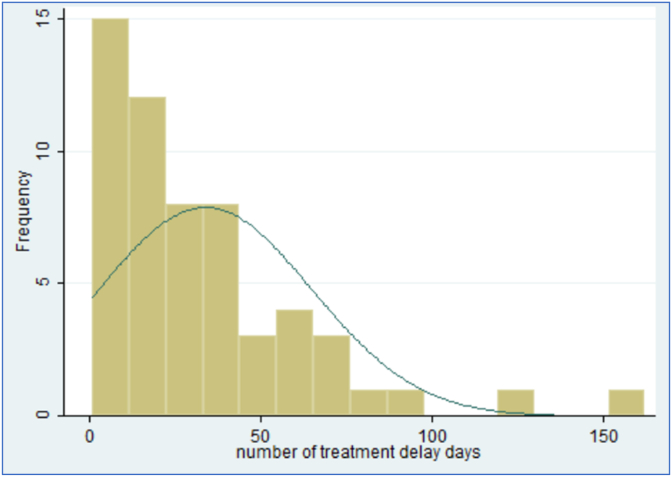
Showing the distribution of days delayed beyond 62 days.

**Fig. 2:**
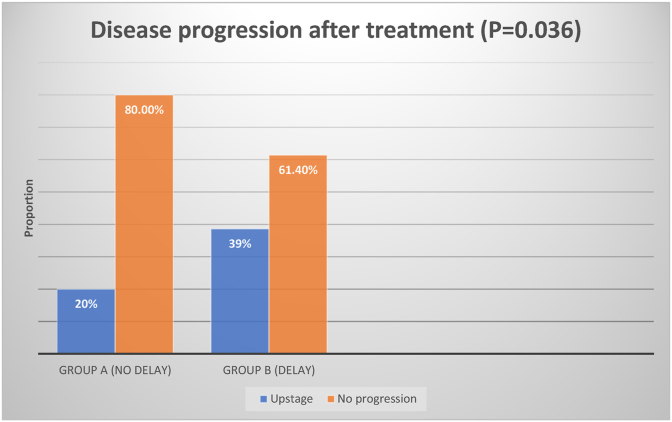
Disease progression after treatment.

The mean wait days for treatment for all patients was 72.7 days ± 34.5 during the COVID pandemic. The median was sixty four days. Fifty (46.7%) patients did not experience treatment delays of more than 62 days. These patients received treatments 46.3 ± 11.5 days from the date of referral compared to 95.8 ± 31.0 days in the group with delay in treatment (p < 0.0001). Only 20% of patients in the group without delay had disease progression by the date of first treatment, whereas 38.6% [22] patients in the delayed group had upstaging of their tumour by their first treatment (p = 0.036) (Fig. 1, Fig. 2).

More patients with rectal cancers had a delay in treatment compared to those with colon cancers (combination of left and right colon cancers) but this difference was not statistically significant (p = 0.860). Patients with rectal cancer in the delayed group waited an average of 38.6 (2.3) extra days compared to 31.6 (3.62) days in colon cancer patients but this difference was also not significant. (p = 0.4408). Patients who upstaged in the delayed treatment group had average delays of 42.2 (39.6) days beyond the sixty two day threshold required by the NHS; compared to 28.5 (23.3) days in those who did not upstage. This difference of fourteen days was however not statistically significant (p = 0.11).

Patients who had a delay in treatment were 3.3 times more likely to upstage than that those who did not have a delay beyond the threshold (95CI: 1.15–9.32; p = 0.026). None of the covariates (BMI, age category, gender, alcohol, and smoking history) were found to be statistically significant (see Table 2).

**Table 2:** **Logistic Regression reviewing delay beyond 62 days**.

Variables	Odds Ratio	P-value	95% CI
Delay category	3.27	0.026	1.15, 9.32
Age Category	0.85	0.771	0.29, 2.48
Male	0.55	0.266	0.19, 1.56
BMI	1.64	0.393	0.53, 5.14
Alcohol intake	0.80	0.662	0.29, 2.21
Smoking	0.71	0.504	0.26, 1.95

The risk of upstaging of cancer increased by 1.5% for every day that a patient's treatment was delayed from the date of referral (Odds ratio: 1.016; 95CI: 1.002–1.030; p-value = 0.020). Included covariates were not statistically predictors of upstaging (Table 3).

**Table 3:** Logistic regression reviewing from the date of referral.

Logistic Regression	Odds Ratio	P value	95% Confidence Interval
Date from referral to treatment	1.017	0.020	1.002, 1.030
Age Category	0.898	0.842	0.316, 2.560
Male	0.429	0.097	0.158, 1.167
BMI	1.343	0.610	0.432, 4.176
Cons	0.162	0.005	0.046, 0.580

## 4 Discussion

This study assessed the effect of treatment delays on colorectal cancer progression in patients at one of the hospitals in the United Kingdom. NHS guidelines released in March 2020 during the pandemic stated that colorectal treatment delays of 10–12 weeks was safe and without likely impact on the outcome for patients. Our study's results however show an impact on outcomes when treatments are delayed beyond sixty-two days. We found that patients with non-metastatic colorectal cancer who had delays beyond 62 days were 3.3 times more likely to experience cancer upstaging compared to those who did not experience treatment delays. We also found that each day of delay after the date of referral was associated with a 1.5% increase in the likelihood of upstaging.

Several other studies have failed to demonstrate that treatment delays influence the progression of disease and survival [22,23,27,28,[37], [38], [39]]. Reasons suggested for the lack of findings include the fact that progression from adenomas to cancer takes between 5 and 15 years with the symptomatic phase being a very late event in the natural history. As such, one to three months delay make little or no difference in outcomes [29]. Also, differences in the biology of the cancers (i.e some cancers are faster growing) may be more responsible for upstaging [40] than delays in treatment after suspected diagnosis. More studies including systematic reviews and meta-analysis are needed to determine the true relationship between delay in treatment and upstaging in tumours.

Our study had several important strengths. Clinical records were used to collate the data, providing real-world evidence on the impact of treatment delays on the progression of colorectal cancer. The high degree of completeness of patients’ records allowed robust adjustment for potential confounders example age and gender. Study limitations include the small sample size used in the study. Our sample size was limited by adhering to the number of patients admitted through the 2-week wait referral system in the two hospitals during the period of the pandemic and because the study period was limited to one year (the beginning of the pandemic).

## 5 Conclusion

Cancer care in the UK has been significantly impacted by the COVID-19 pandemic. Current guidelines suggest that treatment delays of up to twelve weeks from suspected diagnosis do not have significant impact on disease outcomes. But this study demonstrated that delays could lead to a three times likelihood of colorectal cancer upstaging. Our study also showed that only 46.7% of the patients in the NHS trust had their first cancer treatments within 62 days of referral as mandated by the NHS. This proportion is significantly lower than the 85% compliance target set for the trust by National Health Service (NHS) [41].

Despite the ongoing pandemic, cancer treatment should continue to be prioritized because prognosis and survival are closely linked to timely treatments [42]. Manpower, care delivery research and training need to be redirected to cancer care to ensure to ensure better outcomes.

## Ethical approval

Not required.

## Sources of funding

None.

## Author statement

Omotara Kafayat Lesi-Conceptualization, study design, data collection, data analysis, writing, editing.

Ebuwa Igho-Osagie-methodology, data analysis, editing, proof-reading.

Sarah-Jane Walton-editing, supervision.

## Research registration Unique Identifying number (UIN)

1. Name of the registry: Research Registry.

2. Unique Identifying number or registration ID: researchregistry7302.

3. Hyperlink to your specific registration (must be publicly accessible and will be checked): https://www.researchregistry.com/browse-the-registry#home/

## Guarantor

Ms Sarah-Jane Walton- Consultant Colorectal Surgeon, Mid and South Essex NHS Foundation Trust, Basildon and Thurrock University Hospital, United Kingdom.

## Provenance and peer review

Not commissioned, externally peer-reviewed.

## Declaration of competing interest

None.
